# Phase controlled green synthesis of wurtzite (*P*63*mc*) ZnO nanoparticles: interplay of green ligands with precursor anions, anisotropy and photocatalysis†

**DOI:** 10.1039/d3na00596h

**Published:** 2023-11-17

**Authors:** Lahur Mani Verma, Ajay Kumar, Aejaz Ul Bashir, Upanshu Gangwar, Pravin P. Ingole, Satyawati Sharma

**Affiliations:** a Centre for Rural Development & Technology (CRDT), Indian Institute of Technology Delhi Room No. 289, Block-III, Main Building, Hauz Khas New Delhi-110016 India satyawatis@hotmail.com +91-11-26591121 +91-11-26591116; b Department of Chemistry, Indian Institute of Technology Delhi New Delhi India

## Abstract

Green approaches for nanosynthesis often lack the precise control of synthetic outcomes, which is primarily due to the poorly defined reaction protocols. Herein, we investigated the use of lignocellulosic agro-waste, sugarcane press mud (PM), for the synthesis of ZnO nanoparticles using three different precursor salts and their further application in the photocatalytic degradation of rhodamine dyes. This approach resulted in the formation of ZnO nanoparticles with two different morphologies, *i.e.*, sheet-like structure from the zinc sulphate and nitrate precursors, whereas sphere-like structures from zinc acetate. In all three cases, the wurtzite phase (*P*63*mc*) of ZnO nanoparticles remained consistent. Also, the ZnO nanoparticles were found to be positively charged (“*ζ*” = +8.81 to +9.22 mv) and nearly monodispersed, with a size and band gap in the range of ∼14–20 nm and 3.78–4.1 eV, respectively. Further, the potential photocatalytic activity of these nanoparticles was investigated under direct sunlight. At the same photocatalyst dose of 0.1 g L^−1^, the three ZnO nanoparticles showed varying efficiencies due to their shape anisotropy. The ZnO NPs from acetate salt (∼20 nm, sheet like) showed the highest dye degradation efficiency (90.03%) in 4.0 hours, indicating the role of the catalyst–dye interface in designing efficient photocatalysts.

## Abstract 2

Agro-waste based green synthesis of ZnO nanoparticles and sun light driven photocatalysis.

## Graphical abstract

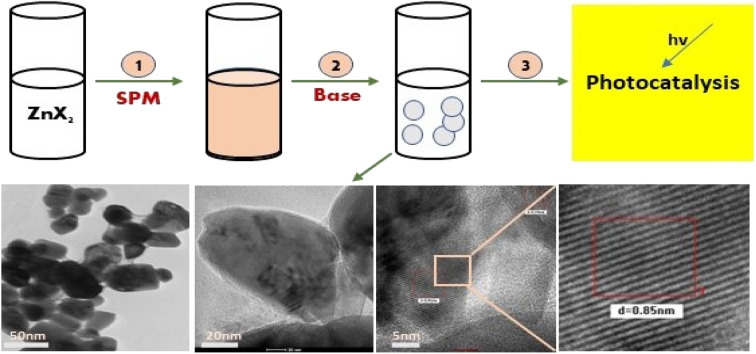

## 1 Introduction

In the ever-evolving field of nanotechnology and the quest for sustainable and eco-friendly solutions, the precise control of the properties of nanoparticles remains an important goal.^1^ In this case, among different nanomaterials, zinc oxide (ZnO) has emerged as a superior material, attracting attention from researchers due to its multifaceted applications in medicine, organic synthesis, electronics, optoelectronics, and photocatalysis.^2–6^ Of particular interest is the wurtzite phase of ZnO, which is characterized by a hexagonal symmetry in the *P*63*mc* space group, which endows exceptional structural and electronic attributes.^7^ As the scientific community advances towards greener and more sustainable practices, the development of eco-friendly strategies for the synthesis of wurtzite ZnO nanoparticles with tunable band gaps has become the focus of research. The green synthesis of nanoparticles, which is facilitated by environment friendly ligands, not only aligns with sustainable principles but also holds potential for the precise fabrication of nanomaterials with required properties.^1^ To achieve this, the preparation of composites and alloys, doping, defect engineering, and the control of morphology and crystallite size are some frequently employed methods. Besides, the recent application of the computational design of materials has accelerated the exploration of configurational and compositional space related to their statistical behaviour at the molecular and atomic scales.^8–10^ Moreover, an understanding of the intricate interplay among precursor materials, reaction conditions, and stabilizing agents can facilitate the fine-control of the synthetic outcomes, but it is not fully understood by now. Among these factors, the utilization of green ligands has emerged as a promising avenue. Green ligands, which are often derived from renewable resources, in this case, agro-waste sugarcane press mud, are rich in various chemicals and play a pivotal role in enhancing the stability of ZnO nanoparticles, while simultaneously influencing their structural, morphological, and functional characteristics.^11–13^ However, most reports focus on the fine-control of uniform morphology and other properties using fine chemicals and advanced techniques.^7,14,15^ Accordingly, in the quest for the synthesis of environment friendly nanomaterials, in this study, we aimed to reveal the intricate relationships among green ligands, precursor anions, and the anisotropy inherent to wurtzite ZnO nanoparticles. The wurtzite phase of ZnO is characterized by anisotropic growth, where preferential crystallographic directions dictate its morphology and arrangement of atoms.^16,17^ This anisotropy holds the key to unlocking the full potential of ZnO nanoparticles in photocatalytic applications.^18,19^ Given that ZnO nanoparticles have different exposed crystal facets, each facet possesses unique surface energies and reactivity, significantly influencing their photocatalytic activity.^20^ Understanding the origins of this anisotropy is fundamental for tailoring ZnO nanoparticles for diverse applications. It also promises to shed light on the design and synthesis of nanomaterials with enhanced performance.^6,18,20^ The application of ZnO nanoparticles in photocatalysis is of particular significance, given their potential to harness solar energy for environmental remediation, namely, fertilizer applications through soil. The wurtzite phase, characterized by inherent anisotropy and surface structure, represents an ideal platform for efficient photocatalytic processes. Thus, by elucidating the mechanisms governing anisotropic growth, this study aimed to advance the understanding of the suitability of ZnO nanoparticles for photocatalytic applications, offering solutions to pressing challenges in the environment and precision agriculture.^20,21^

Herein, we extensively explored the interplay between green ligands and precursor anions in the synthesis of wurtzite ZnO nanoparticles. We focused on the intricate interplay of eco-friendly ligands, uncovering their roles in nucleation, growth, and phase control. We scrutinized how these roles affect the anisotropic characteristics of nanoparticles and explored their broader implications in photocatalytic performance in the environmental and agricultural fields. Through the synergistic interplay of green ligands and precursor anions, we attempted to unlock the full potential of wurtzite ZnO nanoparticles, ushering in a new era of sustainable materials and technologies.

## 2 Materials and methods

### 2.1 Procurement of reagents and other materials

Analytical grade NaOH and H_2_O_2_ (30%) were obtained from Merck (Merck, Germany) and zinc salts (ZnSO_4_·7H_2_O, Zn(OAc)_2_·2H_2_O, and Zn(NO_3_)_2_·6H_2_O) and Rhodamine 6G dye were obtained from Sigma Chemicals (USA) and used as received without further purification. All the reagent solutions were prepared in double-distilled water and the glassware used in the experiments were properly cleaned and dried in a hot-air oven at 180 °C for 2 h before use. Sugarcane press mud generated from the filtration of sugarcane juice was procured from TR Solvents Pvt. Ltd.

### 2.2 Sugarcane press mud analysis

An aqueous extract of SPM was prepared following the protocol reported in ref. 22. It was analysed further using the LC-MS, FTIR, and H^1^ NMR techniques. The LC-MS analysis was performed using a 0.2 μm filtered sample and positive scan (ES mode) on an LC-MS instrument (Agilent Technologies, USA). The H^1^ NMR study was done using a 400 MHz instrument (Ascend 400 Bruker). The functional groups were analysed on an FTIR spectrophotometer (PerkinElmer 1600) in transmittance mode.

### 2.3 Synthesis and characterisation of ZnO NPs

ZnO NPs were prepared following the protocol reported by Doan Thi *et al.* (2020),^23^ with slight modifications. Briefly, a fresh 200 mL solution of 0.1 M ZnSO_4_·7H_2_O was prepared using distilled water and stirred for 10 min at 750 rpm on a magnetic stirrer (Remi 5 MLH Plus), resulting in the formation of a clear solution with pH of 4.23 at 32 °C. To this solution, 15 mL of SPM extract was added slowly, and the final pH of the solution was recorded as 6.02. This solution with extract was left stirring at 750 rpm for another 15 minutes. To this faint-brownish solution, freshly prepared 10 mL NaOH (2.0 M) solution was added gradually to raise the pH of the solution to 12 at 32 °C, resulting in precipitation. The precipitated whitish colloidal solution was further stirred for 20 min, and the solution was filtered using Whatman Filter Paper No. 1 (pore size 11 μm). The air-dried residue was calcinated at 85 °C in a vacuum oven at reduced pressure for 24 h. The same procedure was repeated for the other two precursor salts, *i.e.*, ZnSO_4_·7H_2_O and Zn (OAc)_2_·2H_2_O. The dried samples were further used for characterisation and experimental study. A summary of the synthesis conditions is presented in ESI Table A1.†

**Fig. 1:**
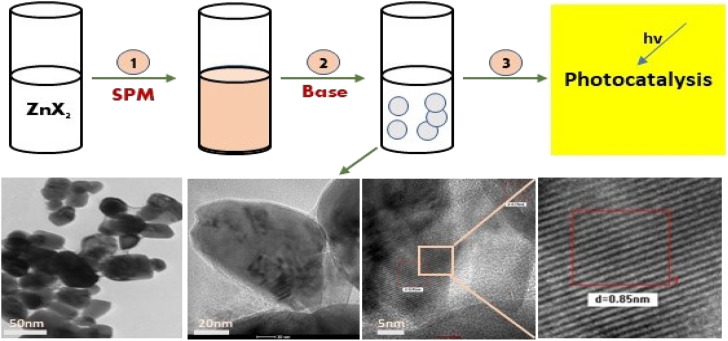
A schematic diagram representing the green synthesis of ZnO NPs and their photocatalytic application.

**Fig. 2:**
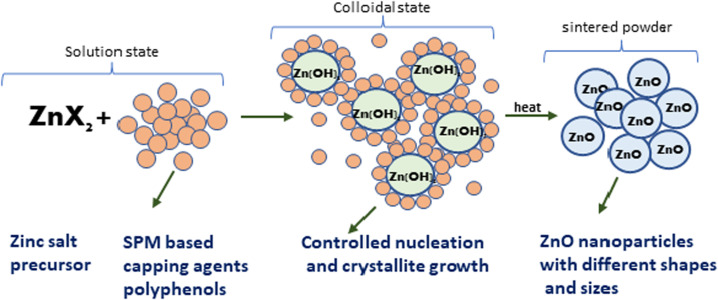
A probable mechanism for the formation of ZnO NPs through the observed stages during their green synthesis.

Further, the characterisation study of the synthesised nanoparticles was performed following the protocol reported by Singh *et al.* (2022).^24^ The phase and crystal structure of the synthesized zinc nanomaterial were determined by X-ray diffraction (XRD) in the 2*θ* scanning range of 20–80° using an XRD X'Pert Pro (PANalytical, Netherlands) diffractometer with a Cu-Kα X-ray source (*λ* = 1.5406 Å). SEM-EDX (scanning electron microscopy with Oxford-EDX system IE 250 X Max 80, Netherlands) and TEM (JEOL JEM-1400) at an accelerating voltage of 100 kV and HR-TEM (Tecnai G2 20) were performed for morphology analysis. The surface of the NPs was analysed using an FTIR spectrophotometer (PerkinElmer1600) in the spectral range of 4000–500 cm^−1^. The UV-Vis absorption study was performed using an Epoch 2 microplate reader (scanning range of 300–700 nm) (BioTek Instruments). Dynamic light scattering (DLS) (Zetasizer Ver. 7.11, Malvern Instruments) was used to estimate the hydrodynamic diameter (HDD) and zeta (*ζ*) potential of the synthesized ZnONPs.^25^ A schematic diagram of the green synthesis and probable mechanism is described in Fig. 1 and 2, respectively.

### 2.4 Electron impedance and cyclic voltammetry

All the ex situ electrochemical measurements were carried out using an electrochemical workstation (Metrohm Autolab 302) in a custom-designed two-chamber three-electrode setup, with one-half cell having a GC drop-casted with catalyst as the working electrode, saturated Ag/AgCl, Cl^−^ as the reference electrode, and *E*^0^ = 0.197 V *vs.* RHE and another half-cell containing Pt mesh as the counter electrode, and the two half-cells were connected by a Nafion proton exchange membrane. About 2 mg of catalyst was dispersed in 100 μL isopropyl alcohol (IPA) with 20 μL 5% Nafion as the binder and drop-casted on a 2 × 2.5 cm^2^ glassy carbon electrode, and then left undisturbed for about 15 min. The electrochemical investigation was carried out in an aqueous KCl solution (pH = 7). All potentials are reported with respect to the reversible hydrogen electrode (RHE) using the following equation:

1 *E*_RHE_ = *E*_Ag/AgCl,Cl^−^_ + 0.197 + 0.059 (pH)

The *C*_dl_ was calculated using the cyclic voltammograms and the following equation:2
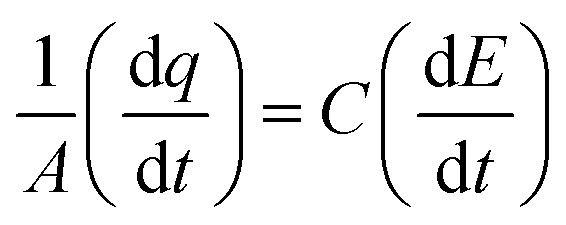
where 
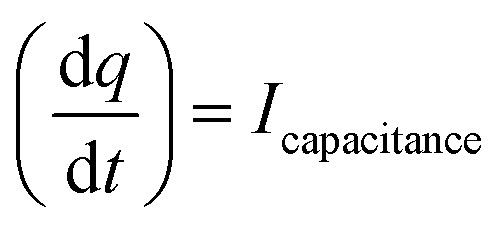
 and 
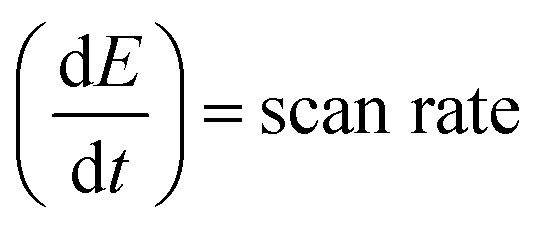
; *A* = geometrical area of the working electrode (W.E) and *C* = area normalized capacitance. 
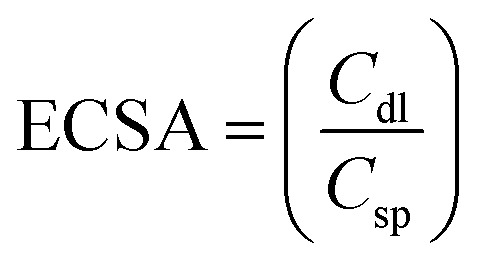
 and *C*_sp_ were assumed to be 20 μF cm^−2^, giving the number of electrochemically active sites per unit area. RF = ECSA/area (W.E) and W.E = 5.2 cm^2^, and this RF gives the number of electrochemically active sites per unit area.

### 2.5 Photocatalytic experiments

The three types of nanoparticles synthesized using zinc sulphate sheet-like 14 nm (Zn-SL), nitrate sheet-like 16 nm (Zn-NR), and acetate sphere-like 20 nm (Zn-AC) were investigated for their photocatalytic activity using dye Rhodamine 6G under varying intensities of direct sunlight (morning 8:30 am to evening 5:00 pm at IIT Delhi, New Delhi, coordinates, 28.5457° N, 77.1928° E India, June 20, average relative humidity 27%, and average day temp. 31 °C). Briefly, 100 mL solution of Rhodamine 6G with a concentration of 0.0596 × 10^−3^ M was prepared separately in a 250 mL beaker using double-distilled water. Then, 5.0 mg zinc oxide nanoparticles was accurately weighed (0.1 g L^−1^) and added to the dye solution (50 mL) and sonicated for 10 min to reach adsorption–desorption equilibrium in the dark, and then 1.0 mL of solution was withdrawn for the initial absorption measurement before exposing the solution to sunlight. After this, the solution was placed in direct sunlight, and the absorption of Rhodamine 6G (*λ*_max_ = 540 nm) was recorded after 30 min intervals using a UV-Visible spectrophotometer (BioTek Epoch2) at a resolution of 1.0 nm. The photocatalytic activity was calculated using the following equation:


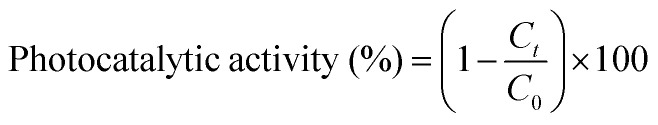
, where *C*_*t*_ and *C*_0_ are the RhB absorbance at 540 nm at time (*t*) = 0 and any time “*t*”. The apparent rate constant (*k*) of the reaction was calculated using the first-order kinetic model to describe the photocatalytic degradation of RhB, which is given by 
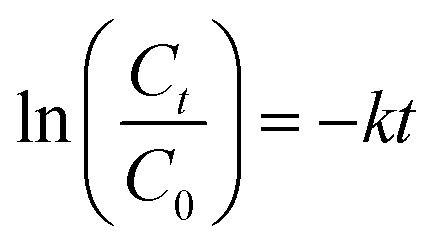
. The cyclic usability test experiment was conducted using the same protocol but a higher concentration, *i.e.*, 45 mg/50 mL.

### 2.6 Hydrogen peroxide (H_2_O_2_) experiment

To investigate the role of OH˙ radical in the degradation of RhB, a hydrogen peroxide (H_2_O_2_) experiment was conducted, following the above-mentioned protocol for the photocatalytic experiment with necessary modifications. A 50 mL dye solution with and without 5.0 mL H_2_O_2_ (30%) was exposed to light after proper mixing. Then, the absorbance of the solution was measured as described above.

### 2.7 Brunauer–Emmett–Teller (BET) analysis

The pre- and post-Brunauer–Emmett–Teller (BET) analysis (N_2_ adsorption–desorption isotherms) to determine the specific surface area (N_2_ gas as the adsorbate) of the catalyst samples degassed at 200 °C, 8.0 h, was done using the BELSORP Data Analysis Software BELMaster Ver 7.2.0.4.

## 3 Results and discussion

### 3.1 Characterisation of sugarcane press mud

The prepared and stored water extract of SPM was subjected to LC-MS, FTIR, and H1 NMR characterisation. To the best of our knowledge, to date, no study has been reported on the analysis of the bioactive components present in the water extract of SPM. Generally, the information available in this regard is limited to the proximate analysis of its physicochemical properties together with its general chemical composition.^13,26,27^ According to these studies, sugarcane press mud contains sugar (5–15%), fibre in the form of cellulose, hemicellulose, and lignin (15–30%), crude wax (5–14%) and crude protein (5–15%) as the organic component with some free functional groups such as aldehydes, ketones, lactones and amines together with hydrocarbons (aromatic and aliphatic) and inorganic salts.^13^ However, in the study by Radwan *et al.* (2021),^22^ in an attempt to recover useful chemicals from sugarcane press mud, reported the recovery of sugar (0.85%) and protein (3.3%) from its aqueous fraction in a pH-dependent manner but there is no further investigation available detailing the recovered metabolites. Moreover, our LC-MS (ESI Fig. S1c and d†) study showed the presence of fragments with an *m*/*z* ratio corresponding to the [M + H]^+^ peak in the range of 148.90 to 735.22 with relatively intense peaks at *m*/*z* 665.17, 679.11, 338.10 and 339.10 having a significantly resolved retention time in the LC (Fig. S1c†) chromatogram. The probable compounds corresponding to these fragments were searched in a mass library database (Metlin) and further validated by the literature.^28,29^ The probable compounds and their functional groups were supported by the corresponding ^1^H NMR (Fig. S1a and b†) chemical shift values and (Fig. 17) characteristic FTIR stretching frequencies in the range of 3300–3550 for (broad) O–H, 2950–2800 for –C–H aromatic; 2000–2100 for aromatic overtone hump; 1580–1600 for –C=C– aromatic; for 1400–1300 –C–H in-plane bending; and 1150–1100 –C–H out-of-plane bending.

### 3.2 Synthesis and characterisation of ZnO nanoparticles

**Fig. 3:**
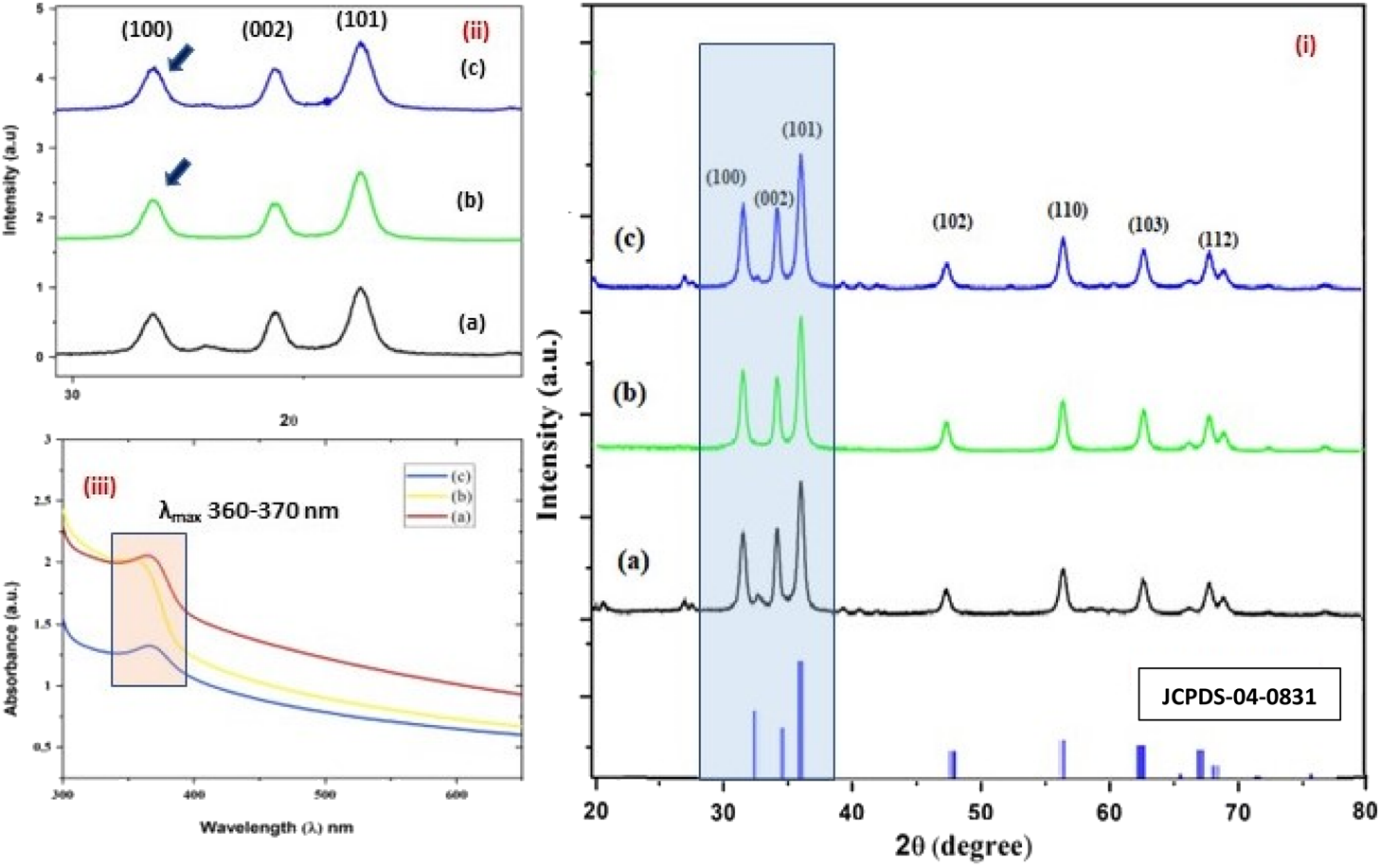
(i–iii) The X-ray diffractograms (i and ii) of the three structurally different NPs of ZnO samples, “*b*” and “*a*” refer to zinc sulphate and zinc nitrate, respectively (sheet-like structure), and “*c*” to zinc acetate having a size of 15 nm, 18 nm, and 20 nm, respectively. The UV- absorption spectra (iii) of the three ZnO NPs having similar *λ*_max_ values, *i.e.* 360–380 nm.

**Fig. 4:**
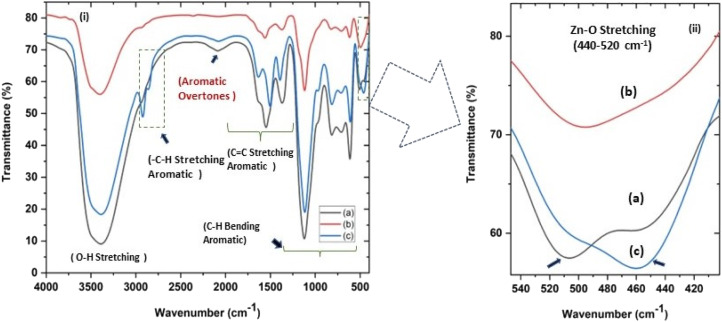
(i and ii) Fourier transform infrared spectra (FTIR) of all three ZnO NPs samples showing (c) a characteristic absorption at around 500 cm^−1^ for Zn–O stretching. (ii) shows (magnified a and c) the splitting of the characteristic Zn–O stretching peak at 451–509 cm^−1^ (a) and 460–506 cm^−1^ (c) for zinc sulphate and nitrate precursors, respectively, indicating sheet-like structures and (b) an unsplit peak at around 494 cm^−1^ for sphere-like ZnO NPs from zinc acetate precursor.

**Fig. 5:**
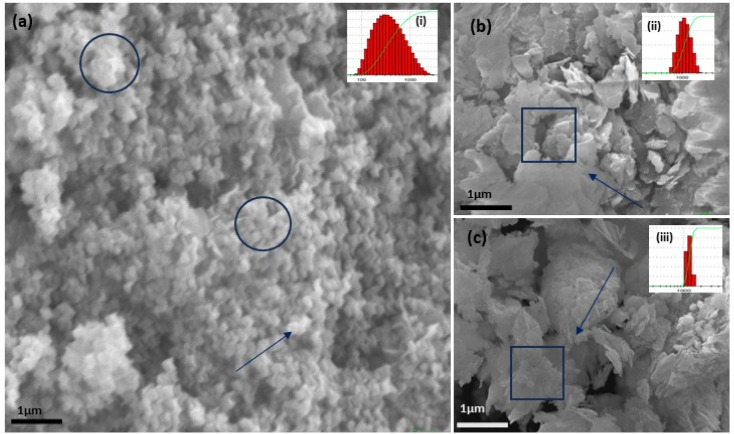
The SEM (a–c) images of the three structurally different ZnO NPs from three different salt precursors, *i.e.*, sphere-like structure from zinc acetate (a) and sheet-like structure from zinc nitrate (b) and zinc sulphate (c). DLS analysis for three samples with varying (but narrow size distribution) PDI of 0.486 for acetate (a), 0.222 for nitrate (b), and 0.277 for sulphate (c) in the top inset figure.

**Fig. 6:**
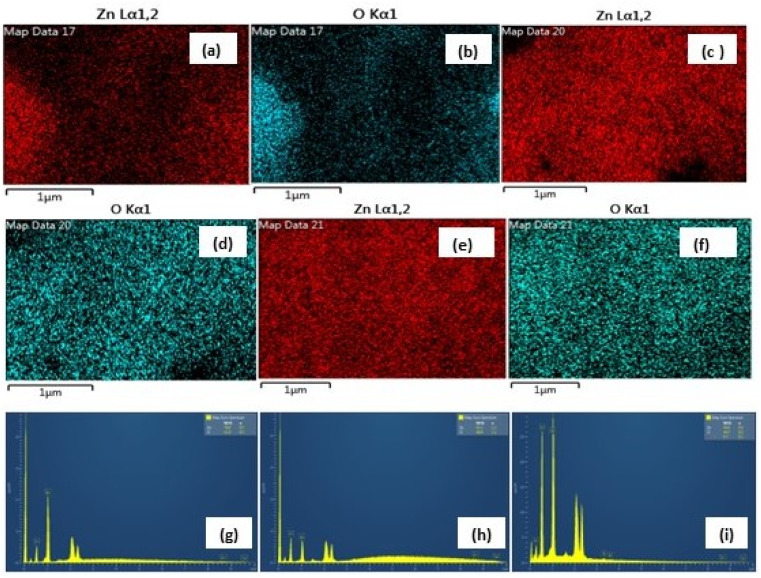
(a–f) The similar EDX mapping analysis of Zn and O (high purity) in the three samples and (g–i) weight% of Zn, 47.95%; O, 52.05% in nitrate (g), Zn, 39.63%; O, 60.33% in sulphate (h) and Zn, 39.58%; O, 60.42% in acetate (i) (inset, bottom).

**Fig. 7:**
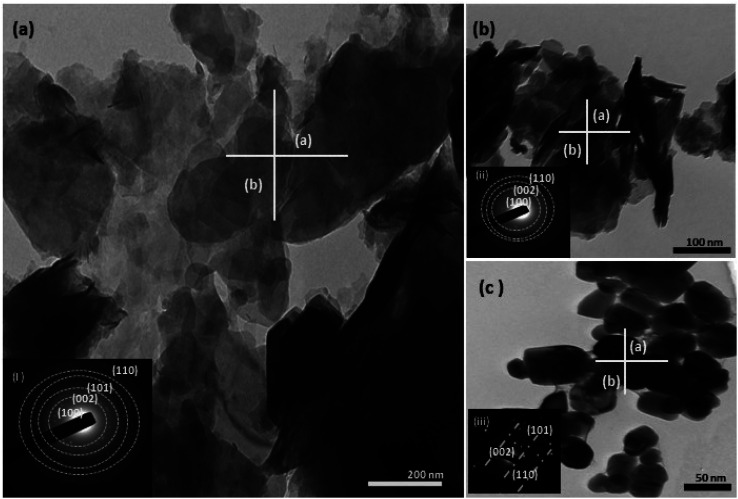
The TEM (a–c) images of the three structurally different ZnO NPs from different salt precursors, *i.e.*, sheet-like structure from zinc nitrate (a), zinc sulphate (b), and sphere-like structure from zinc acetate (c). The corresponding SAED pattern (inset figure) for the three samples shows the polycrystallinity of the samples (a and b), while the SAED pattern for sample (c) shows a shift toward a monocrystalline material.

**Fig. 8:**
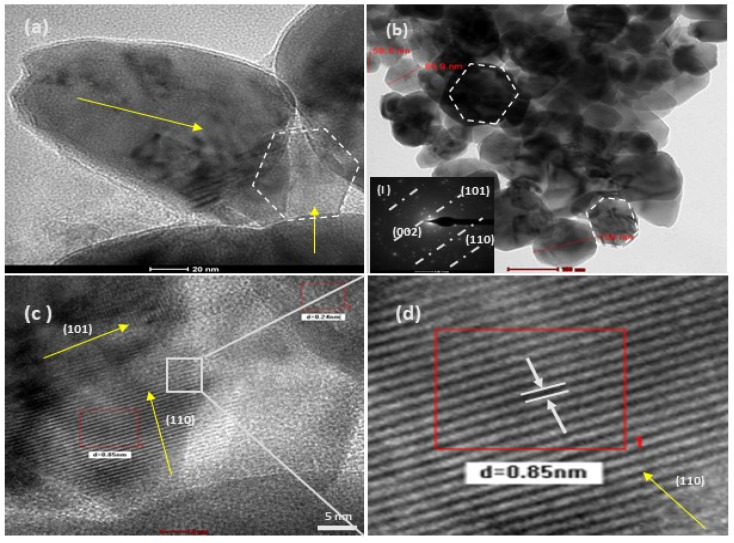
HRTEM (a–d) images of at varying resolutions for the structurally different ZnO NPs from the salt precursor of zinc acetate (4c). Image (b) shows sphere-like NPs, which on higher magnification (20 nm scale), show a slight irregularity in their shape. The image (c) is highly resolved (5 nm scale), and (d) is its selected area magnified image, showing a regular crystal lattice with a *d* spacing of 0.85 nm. The corresponding SAED pattern (inset figure) for this sample shows a shift toward a monocrystalline material.

**Fig. 9:**
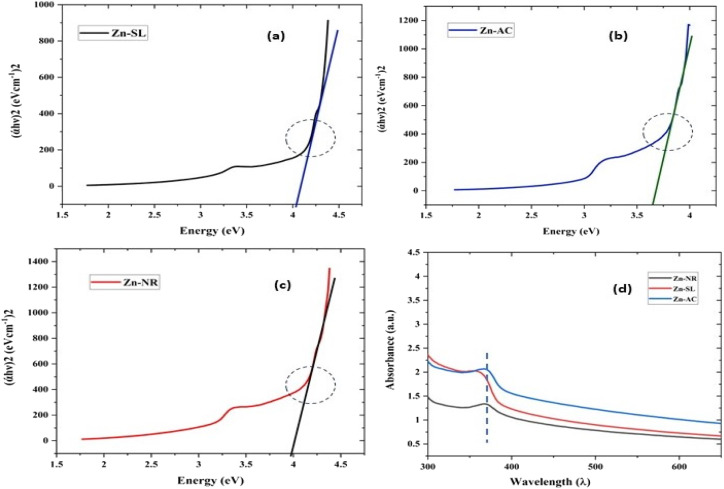
The Tauc plots of the three samples Zn-AC with a band gap of 3.78 eV (a), Zn-SL with a band gap of 3.96 eV (b) and Zn-NR with a band gap of 4.1 eV (c). (d) The UV-visible absorption spectra showing the shift in the absorption edges due to the corresponding quantum confinement of charge in a given dimension of ZnO NPs.

In the present study, the role of the precursor salts (sulphate, nitrate, and acetate salt of zinc) on the shape, size, stability, and yield of NPs was investigated at pH = 12 and reaction temperature of ∼35 °C. The previous work showed the instrumental role of the precursor salt in the size and morphology of nanomaterials under well-defined conditions, which often involve the fixed thermodynamic and kinetic parameters of the systems in use.^30^ Considering the versatility of the ZnO material, there have been efforts to develop methods to fine-tune its crystal structure, morphology, surface, and size. For instance, many studies have shown the use of various nucleating agents, capping agents, precursors, and organic solvents, with diverse dielectric constant (*ε*) and polarity, with an appreciable degree of control on the above-mentioned properties.^31–33^ However, most of these studies have focused on the crystal growth and its regulation by external factors such as solvents, capping agents, and rate of the dissolution of the precursor salt. One common conclusion is the arrest of the axial or planar growth of the ZnO crystal, exploiting the inherent difference in the polarity of its faces and its corresponding surface free energies (Δ*G*), leading to a finite shape.^20,31^ For instance, in the polar wurtzite hexagonal close-packed (hcp) structure of ZnO with the *P*63*mc* space group, where the Zn^2+^ atoms occupy the alternating tetrahedral sites, crystal growth along the crystallographic axis “*c*” perpendicular to the basal plane (001) leads to a rod-shape morphology. In contrast, crystal growth along the plane (100) parallel to the “*c*” axis leads to the formation of a plate-like morphology. Thus, this interplay among the electrical polarity and free energy of the planes and the surfaces with the local environment (solvents, ligands, capping agents, *etc.*) plays a key role in deciding the morphology of nanocrystals. We previously reported the role of ligands covalently ligated to the central metal of the precursor in deciding the morphology of nanocrystals.^34^ However, cases of precursor salts pertaining to the ligands electrostatically attached to the metal center have not been investigated in the required detail. Moreover, a few cases pertaining to this have been reported recently. Specifically, a study demonstrated the varying degrees of screening effects shown by the counter anions of the precursor salts during nucleation and crystal growth, defining the morphology, and thus physicochemical properties of NPs.^35–37^ However, the studies exploring the role of the precursor salts on these properties of NPs have not addressed the combined role of the electrostatically attached counter anions in the precursor salts together with competing external capping ligands. Thus, to further the understanding in this direction, this study investigated the combined role of the counter anions of the precursor salts with external capping ligands on the shape anisotropy and various characteristics of NPs. The experimental results showed that even the stronger screening effects of carbon-based acetate ligands than sulphate and nitrate, as reported by Pourrahimi *et al.* (2014),^30^ could be suppressed in the presence of larger organic ligands (polyphenols and glycol-oligosaccharides) from bio-resources (PM), but in a different manner. This study expands the space for organic ligands in nanosynthesis and furthers the use of agro-industry waste (PM) based on the use of different phytoextracts and various other bio-resource-based materials having common origin to the plants, realising the goal of developing a green approach with fine control. The novel results of these experiments support this hypothesis. Specifically, the TEM (Fig. 7a–c) and SEM (Fig. 5a–c) images reveal the nanosheet-like morphology of the NPs produced using sulphate and nitrate salts of zinc, while sphere-like structure for the acetate salt of zinc with an average diameter of 14, 18, and 20 nm, respectively. This is different from the results shown in the previous studies by *Pourrahimi et al.* (2014),^30^ perhaps because of the absence of any external capping ligands, which direct the crystal growth from the initial stage of nucleation.^30,35,36^ Unlike the studies conducted by Ali and Gates (2008)^35^ and Pourrahimi *et al.* (2014),^30,35^ the results of this study can be explained by considering the superior complexing abilities of the capping agent (polyalcohol) present in the water extract of sugarcane press mud (PM) than the simple electrostatically attached counter anions of the precursor salt. Furthermore, the sheet-like structure (Fig. 5b and c and 7a and b) in the two cases (nitrate “*a*” and sulphate “*b*”) but the sphere-like structure (Fig. 5a and 7c) in the third case (acetate “*c*”) indicate the specific but differential binding abilities of PM-based ligands to a given face of the nano-crystallite during crystal growth.^38^ This is expected based on a previous study that the polar functional groups of the capping agent may have bonded with the polar face of the (001) planes of the wurtzite ZnO nanocrystal, arresting its growth along the crystallographic axis “*c*”, which resulted in a sheet-like morphology. In contrast, in the case of the bidentate acetate (CH_3_COO^−^) ligand, this association may be less probable, resulting in the formation of a sphere-like morphology. This capping ability of PM-based ligands was further supported by the FTIR (Fig. 4i and ii) analysis. The correlation between the characteristic vibrations of the PM water extract (Fig. 9e) and the ZnO NPs (Fig. 4i) confirms the capping of the NPs with the phyto-active components present in the water extract of PM. The common peaks including the broad peak at 3350–3550 cm^−1^ for the O–H stretching from the polyalcohol compounds from the PM water extract, peaks at 2600–2800 cm^−1^ for the C–H aromatic stretching, low-intensity peak at around 2000–2100 cm^−1^ for the aromatic overtones, peaks at 1400–1600 cm^−1^ for the C=C aromatic and intense peak at 1000–1100 cm^−1^ for the C–H aromatic bending indicated the presence of the PM-based ligand on the surface of the NPs.^16,39,40^ In addition, the characteristic stretching vibrations corresponding to the Zn–O bonds at (495–500 cm^−1^) in all three samples is absent in the PM case (Fig. 9e). However, the splitting and shape (Fig. 4iia and b and c) of the characteristic peak of Zn–O at around 500 cm^−1^ confirm the sheet-like structure (sulphate “*b*” and nitrate “*c*”) and sphere-like shape (acetate “*b*”) according to their different axial ratios (*c*/*a*) and shape factors (*g*) based on the theory of average dielectric constant (TADC).^41–43^ The X-ray diffraction (Fig. 3i and ii) studies of all three highly pure (EDX analysis (Fig. 6a–i), samples (nitrate, sulphate, and acetate) demonstrated a polycrystalline phase (SAED pattern, Fig. 5 and 7i and ii) for the sulphate and nitrate case and a shift toward monocrystalline (SAED pattern, Fig. 5 and 7iii) for the acetate salt. The clear concentric SAED pattern in the former two and the spot diffraction pattern in the latter show a shift toward monocrystalline behaviour, which was further confirmed by the HR-TEM images (Fig. 8). The HRTEM images corroborated these findings with the good agreement in interplanar spacing (*d* = 0.85 nm) corresponding to the (110) plane. The average size of the nano-crystallite was estimated using the Debye Scherrer formula (eqn (3)), as follows:3
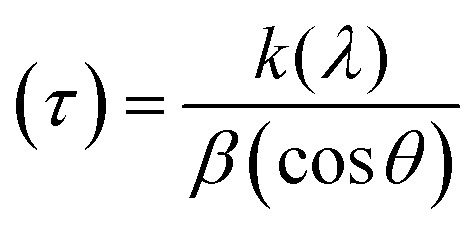
where (*D*) is average crystallite size, *λ* is the wavelength for Cu Kα radiation of 1.5406 Å, *β* is the full width at half maximum (radian) corresponding to the (101) plane, and *θ* is the Bragg angle in degrees).

**Fig. 10:**
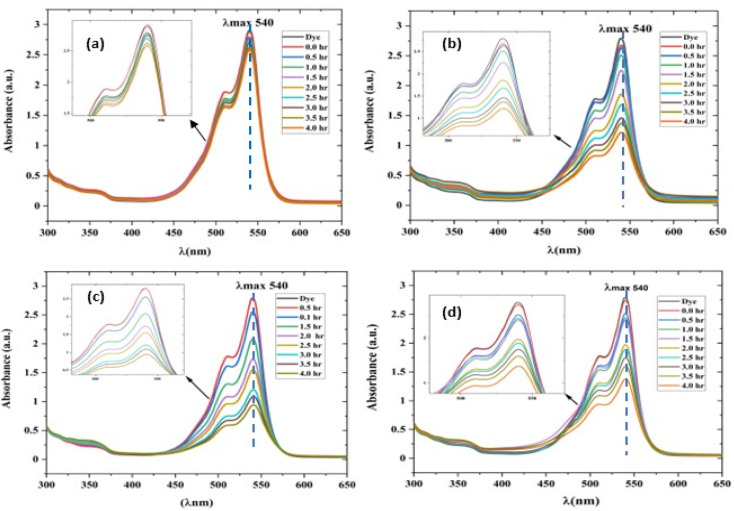
UV-visible absorption spectrum of a rhodamine dye (conc. 0.596 × 10^−3^) and photocatalyst (conc. 100 mg L^−1^) suspension system under exposure to direct sunlight recorded at the time interval of 0.5 h. (a) The control experiment (dye-catalyst system in the dark) and (b) Zn-SL, (c) Zn-AC and (d) Zn-NR catalyst systems.

The average size of 14, 18, and 20 nm was calculated for sulphate, nitrate, and acetate, respectively. The relative intensity ratio of the three peaks corresponding to the Miller indices of *hkl* (100), (002), and (101) (Fig. 3ii) indicates the preferred orientation (non-spherical sheet-like structure) in the case of sulphate “*b*” and nitrate “*c*,” whereas the equal intensity ratio of the (100) and (002) peaks in the case of acetate “*a*” shows no preferred orientation in the sphere-like structure.^42,44^ This again demonstrates the specific but differential capping abilities of the ligands in the presence of structurally different electrostatically attached counter anions in the precursor salts.^30,35^ This is clearly consistent with the FTIR (Fig. 4) results. The reflections were indexed and matched with JCPDS no. 80-0075, which correspond to the lattice planes of wurtzite symmetry with hexagonal cell structures in all three samples. This was also further confirmed by the SEM (Fig. 5a–c) and TEM (Fig. 7a–c) results, respectively. The differing case of acetate (sphere-like structure) was further confirmed by HR-TEM (Fig. 8a–d) imaging, where the lattice fringes show a defect-free nearly monocrystalline lattice with an interplanar spacing of *d* = 0.85 nm. The purity of the synthesized ZnO NPs was studied using SEM EDX. Trace impurities of C, N, and S from the carbon-based grid and precursor salt anion were absent (Fig. 6a–f). The UV-Vis spectra in Fig. 3(iii) indicate that all three samples showed absorption with the *λ*_max_ at 360–370 nm with a clear blue shift compared to bulk ZnO due to the quantum confinement effect at the nanoscale, indicating that the ZnO particles have a size in the nano range.^45^ Further the Tauc analysis (Fig. 10a–c) was performed using eqn (4) (where “*α*” is the absorption coefficient, *h* is Planck's constant 6.626 × 10^−34^ J s, “*ν*” is the frequency of the photon, “*a*” is a proportionality constant, “*E*_g_” is the band gap energy and “*γ*” is the order of the allowed transition and 2 for the direct allowed transition in this case).

4 (*αhν*)^*γ*^ = *a*(*hv* − *E*_g_)

It showed the band gap of 3.78, 3.96 and 4.1 eV for Zn-AC, Zn-SL, and Zn-NR, respectively. This band gap widening compared to the bulk ZnO (3.10–3.70 eV) can be rationalised based on the nano-size effect of the ZnO nanomaterial.^6^ The surface of the fabricated ZnO NPs was found to be positively charged, with the zeta potential (Table 1) recorded for the nanoparticles synthesized from nitrate (Zn-NR) salt (+7.27 mV), followed by that obtained from (Zn-SL) sulphate (+8.81 mV) and (Zn–Ac) acetate (+9.22 mV). The average polydispersity index (PDI) of the NPs (Fig. 5i–iii, inset) followed the trend of nitrate (0.222) < sulphate (0.277) < and acetate (0.486). The unchanged sign of the surface charges in the face of the different counter anions of the precursor salts and size distribution of the ZnO NPs in all three samples reflect the capping ability of the ligands present in the water extract of sugarcane press mud. A summary of the synthetic outcome is present in Table 1.

**Table 1:** The characteristics of ZnO NPs synthesized at pH 12.0 and temperature 35.0 °C

Precursor salts	Morphology (shape)	Size nm	Zeta potential (mv)	Polydispersity index (PDI)	Yield (%)
Zinc sulphate	Sheet like	14	+8.81	0.277	98.6
Zinc nitrate	Sheet like	18	+7.27	0.222	73.4
Zinc acetate	Sphere like	20	+9.22	0.486	60.2

### 3.3 Photocatalytic activity

**Fig. 11:**
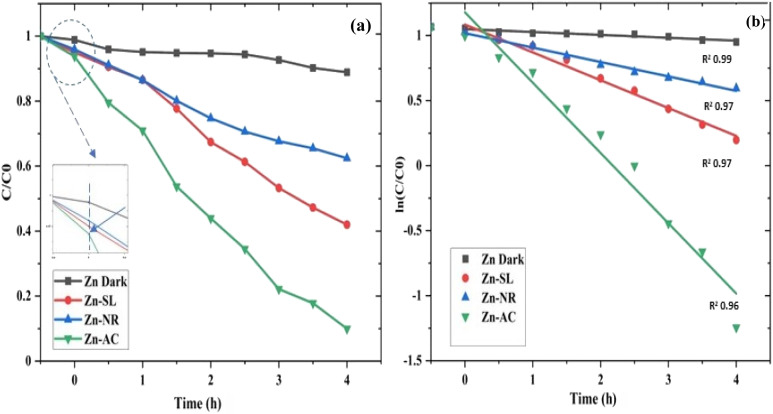
(a and b) A concentration (*C*/*C*_0_) *vs.* time plot (a) showing decrease in dye concentration with time for different catalyst systems and (b) a plot of (ln *C*/*C*_0_) *vs.* time showing pseudo-first-order kinetics with slope (rate constant “*k*”) in *m* = −0.04161 to −0.07473) with *R*^2^ values in the range of 0.95 to 0.99.

**Fig. 12:**
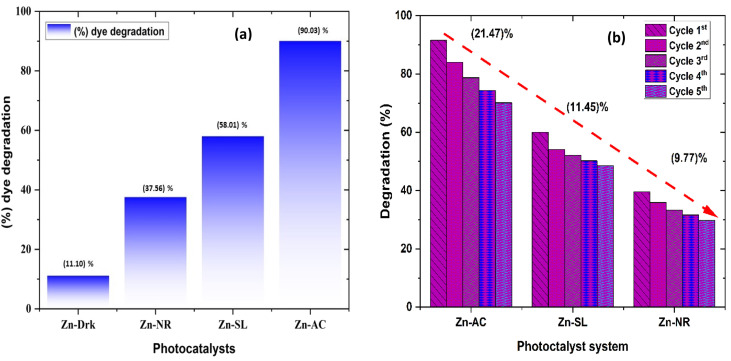
(a) Percentage (%) dye degradation from different catalysts under direct sunlight after 4.0 h and in the dark; (b) percentage (%) decrease in catalytic efficiency of the catalysts over repeated catalytic cycles.

**Fig. 13:**
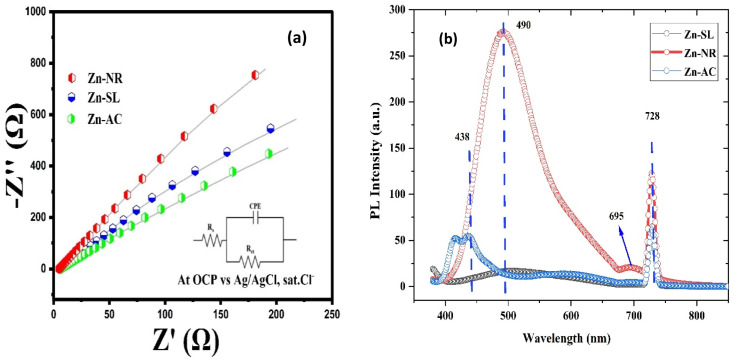
(a) The electrochemical impedance spectra of the Zn-AC, Zn-SL, and Zn-NR catalysts showing different charge transfer resistance (*R*_ct_) at the electrode/electrolyte interface. (b) The photoluminescence spectra of the same catalysts showing their (e^−^) and (h^+^) pair recombination behaviour.

**Fig. 14:**
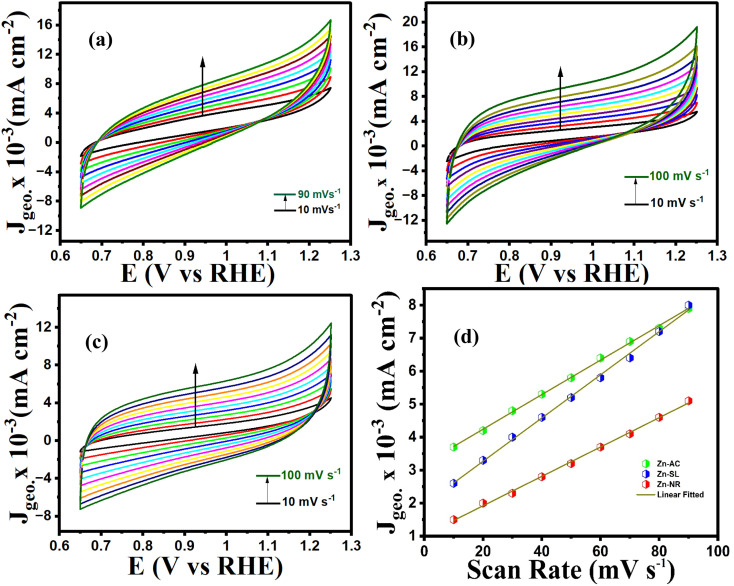
Cyclic voltammograms (10–100 mV s^−1^ capacitive region) of (a) Zn-AC, (b) Zn-SL, and (c) Zn-NR catalysts. (d) *J vs.* scan rate for the calculation of *C*_dl_ (proportional to ECSA) values.

The photocatalytic activity (Fig. 11a–d) of the two structurally similar (Zn-SL and Zn-NR) and structurally different (Zn-AC) nanoparticles was investigated. The experiment was designed to determine the effect of the structural differences in the photocatalysts on their photocatalytic activity for the same rhodamine dye. Considering this, the dye concentration was kept low, *i.e.*, 0.596 × 10^−3^ with a low catalyst loading of 0.1 g L^−1^. This experimental design aimed to decrease the possibility of multilayer adsorption of the dye on the catalyst surface after the adsorption–desorption equilibrium, which will allow the weakening of the concentration effect on the photocatalytic activity of the catalyst.^46^ The result using UV-visible absorption coefficient (*ε*) corresponding to the *λ*_max_ of 540 nm (Fig. 11a–d) showed the significant variation in the degradation percentage of the dye, *i.e.*, only 11.10% in the dark by the Zn-AC catalyst, while 37.56% by Zn-NR, 58.01% by Zn-SL, and 90.03% by Zn-AC (Fig. 12a) catalysts under light conditions at the completion of 4.0 h. The first-order rate constant “*k*” (min^−1^) was observed to vary in the range of 0.04161 to 0.07473 for these photodegradation processes. However, this study is typically done with a low catalyst loading compared to previous studies, and thus a separate experiment with a high catalyst loading, *i.e.*, 0.9 g L^−1^, was performed, which showed (Fig. S2 and S1†) very high rates constants “*k*” (Table S1†) in the range of 0.28606 to 1.81453 min^−1^ with nearly 100% RhB degradation in just 75 min by the Zn-AC catalyst, which is higher rate (Table 3) than that in previous studies.^47^ The large difference in the degradation percentage (rate constant “*k*”) under dark and light conditions indicates the underlying fundamental mechanism of dye degradation, where the initiation of the process as the first step is the absorption of light by the catalyst for the generation of charge carriers, *i.e.*, electron–hole pairs.^48^ On the contrary, the 10% degradation of the dye under dark conditions may be due to the residual absorption of light during the whole experiment. However, the differences in the degradation percentage of the dye due to the different catalysts is primarily due to the morphological differences in the catalysts.^20,31,33,34,49–51^ This is evident by the fact that the Zn-NR and Zn-SL catalysts are morphologically similar, having ZnO nanoparticles with a sheet-like structure (14–16 nm) of the wurtzite phase, while the Zn-AC catalyst has ZnO nanoparticles with a sphere-like structure of the same phase wurtzite. Given that the surface area and the active sites of the catalysts are different, as shown by the BET analysis (Fig. 16) and cyclic voltammetry curve (Fig. 14), due to the structural and geometrical differences of the catalysts, different degrees of dye adsorption occurred on the surface of the catalyst.^46^ This was also evidenced by the shift in the UV absorption in terms of the molar extinction coefficient (*ε*) value (Fig. 11a). The smaller difference in the UV absorption shift (Fig. 11a) in the case of Zn-SL and Zn-NR, while the slightly higher difference in the case of Zn-AC after adsorption–desorption equilibrium indicate the differences in the extent of adsorption of dye on the catalyst surface primarily due to the different morphologies of the catalysts or the polar (002) or non-polar (100) facets of the wurtzite (*P*63*mc*) crystals, as reported by Kislov and Mclaren *et al.*^20,47,52^ This observation is also consistent with the pseudo-first order kinetics of dye degradation (Fig. 11b) with a nearly linear fit (*R*^2^ of 0.95–0.99) of the time (*t*) *vs.* ln(*C*/*C*_0_) curve. Moreover, this large difference in the degradation percentage can also be due to some other factors because the ultimate degradation is the total of various other factors such as the efficiency of light absorption by the catalyst, followed by the efficiency of charge transfer on the surface or other factors.^20,31,48^ Although the catalysts exhibit a small difference in size, and thus the band gap energy accounts for the extent of surface adsorption of the dye and light absorption by the catalyst, the role of the rate of the generation, migration, and recombination of electron–hole pairs remained noticeable. Beyond the similarity of the crystal lattices, *i.e.*, wurtzite in the three ZnO NPs, the defects in the crystal affected the (e^−^)–(h^+^) recombination rates significantly. This, together with the charge transfer resistance (*R*_ct_) across the catalyst–dye interface increase the difference in the degradation efficiency of the three catalysts. The room-temperature photoluminescence (Fig. 13a) and electron impedance spectroscopy (Fig. 13b) results corroborated this,^48^ which can be attributed to the collective effects due to the differences such as catalyst morphology, exposed set of crystal planes, polarity, specific surface area, defects, and the barrier to interfacial charge transfer efficiency. However, the exact cause should be further investigated under suitable experimental conditions. Nevertheless, the results of these experiments demonstrate the role of the interface in efficient photocatalytic degradation reactions.

### 3.4 Electron impedance and cyclic voltammetry

To further investigate the observed differences in photocatalytic efficiency of the three catalysts, electron impedance measurement was performed (Fig. 13b), which showed the varying degrees of charge transfer resistance (*R*_ct_) in the three catalysts. The catalyst system with the lowest rate of degradation (Zn-NR) showed the highest charge transfer barrier in the EIS spectrum and *vice versa* for the Zn-AC system, explaining the difficulty in interfacial charge transfer, as stated above. This is also consistent with the intrinsic or exchange current density (*J*_o_) and standard rate constant (*k*^o^), *i.e.*, the measure of the inherent rate of electron transfer across the interface. This was further corroborated by the highest (Zn-AC) and lowest (Zn-NR) BET-specific surface area (g cm^−2^) for dye adsorption, as show in Fig. 16. In addition, the electrochemically active surface area (ESCA) was measured by cyclic voltammetry (CV) (Fig. 14 and Table 2), which is related to the charge transfer activities for the three catalyst systems, respectively. The cyclic voltammetry (CV) diagram obtained in the capacitive region shown in Fig. 14 indicates the higher current density (Fig. 14a–c) and high *C*_dl_ value (Fig. 14d) for the Zn-AC system, confirming the higher density of active sites in this catalyst towards electron transfer, which demonstrates its highest activity among the catalysts. The ECSA ratio was determined to be 1.5 : 1.2 : 1 for Zn-AC : Zn-SL : Zn-NR. Similar findings have been reported in previous studies.^53,54^ However, as stated earlier, the difference in photocatalytic ability is not ascribed to this. The photoluminescence (PL) spectra (Fig. 13b) also showed the highest recombination rate for the Zn-NR system, which explains the intrinsic difference of the material due to its geometry and defects. In summary, the interfacial activity of all these catalyst–dye interfaces is a function of multiple parameters, which should be considered in the design of efficient catalyst systems.

**Table 2:** The *C*_dl_ values of catalysts calculated from CV curves

Catalyst	*C*_dl_ (μF cm^−2^)	ECSA	RF (cm^−2^)
Zn-AC	655	32	6.2
Zn-SL	523	26	5.0
Zn-NR	455	22	4.2

**Table 3:** A comparison of the rate constant of nano ZnO catalyst systems for the degradation of a RhB dye

S. no.	Reference	Rate constant (min^−1^)
1	This work	0.07473
2	Tuc Altaf *et al.* 2023 (ref. 47)	0.0356
3	Lim *et al.* 2021 (ref. 57)	0.0542
4	Alvi *et al.* 2017 (ref. 59)	0.0246
5	Nandi *et al.* 2019 (ref. 55)	0.042
6	Kumar *et al.* 2021 (ref. 60)	0.0039

### 3.5 Hydrogen peroxide (H_2_O_2_) experiment

**Fig. 15:**
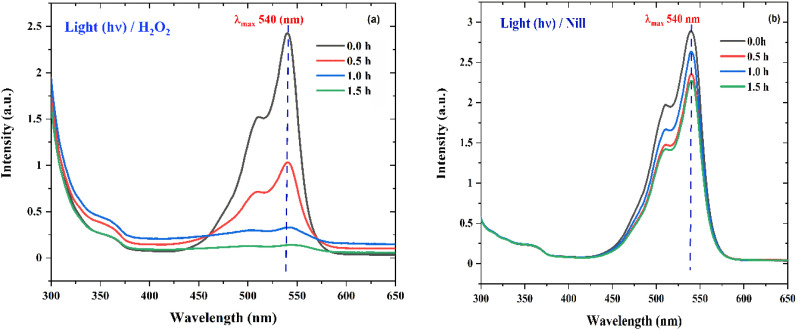
(a) The UV absorption degradation curves of RhB dye with Zn-AC catalyst suspension system for the hydrogen peroxide (H_2_O_2_) experiment in the presence of light (*hv*). (b) The UV absorption degradation curve of RhB dye with Zn-AC catalyst suspension system for hydrogen peroxide (H_2_O_2_) control experiment (without H_2_O_2_ in the presence of light (hv).

To probe the probable mechanism and pathway for the degradation of RhB by the nano ZnO catalysts, an H_2_O_2_ experiment was performed, wherein 50.0 mL dye of the same concentration containing 5.0 mL H_2_O_2_ (30%) under direct sunlight showed nearly 100% degradation (Fig. 15a) of dye in 1.5 h, while the control reaction (only 50.0 mL dye solution) showed (Fig. 15b) no activity. This clearly indicates that in the absence of the catalyst, the necessary OH˙ radicals leading to the degradation reaction were supplied through homolytic fission of the peroxide linkage in H_2_O_2_ by the sunlight.^47,55–58^ The proposed mechanism for the degradation process is given below.^56^

5 ZnO + *hv* (sunlight) → ZnO (h^+^) + ZnO (e^−^)

6 ZnO (h^+^) + H_2_O → OH˙ + H^+^

7 ZnO (e^−^) + O_2_ → O_2_˙^−^

8 O_2_˙^−^ + H_2_O → OOH˙ + OH^−^

9 OOH˙ + H_2_O → OH˙ + H_2_O_2_

10 H_2_O_2_ + *hv* → OH˙ + OH

11 OH˙ + RhB (dye) → CO_2_ + H_2_O

### 3.6 Cyclic reusability test

**Fig. 16:**
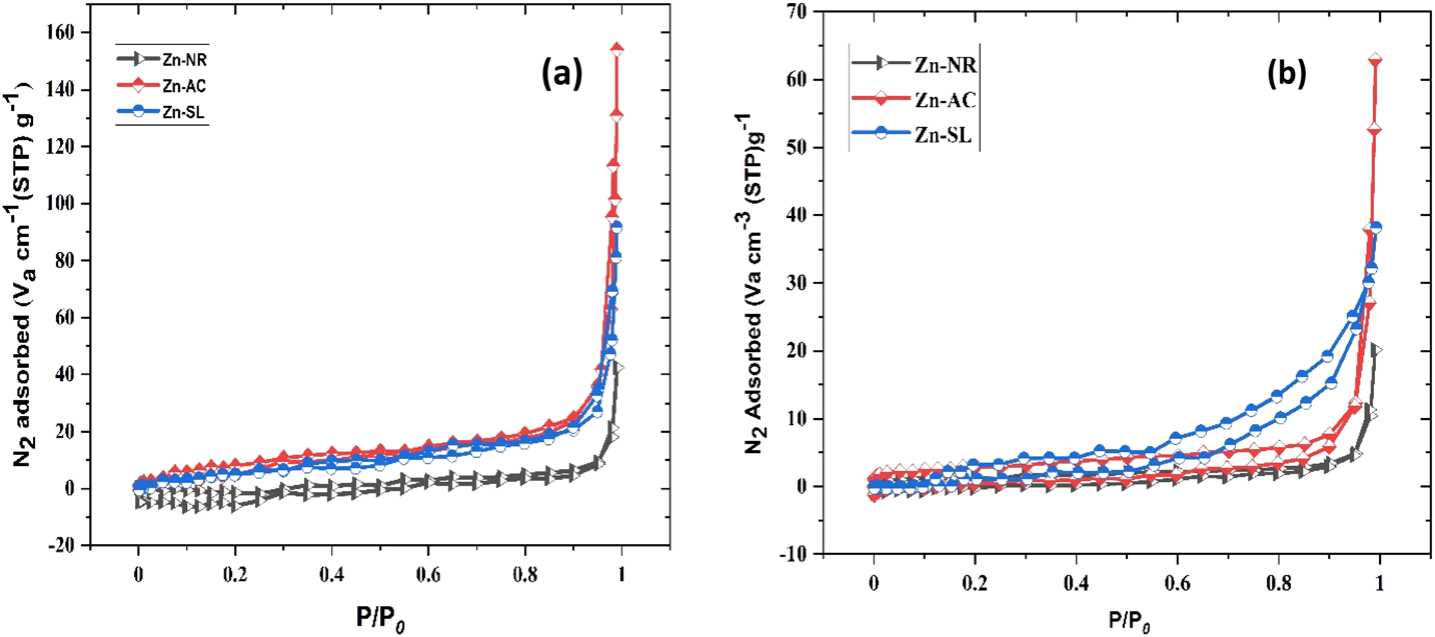
(a) N_2_ adsorption–desorption isotherm of the three catalysts used (Zn-AC, Zn-SL, and Zn-NR), indicating the changes in the surface area of the catalysts (pre- and post-use) by Brunauer–Emmett–Teller (BET) analysis (before use) and (b) isotherm curves after the use of the catalysts.

The photocatalytic cycle was repeated five times. The catalyst was recovered from the suspension by filtering using Whatman filter paper (125 mm), followed by washing using distilled water and air drying in the dark. The catalyst was found to be stable over repeated cycles. However, a slight overall decrease (Fig. 12b) in the catalytic efficiency was noted, *i.e.*, 21.47% for Zn-AC, 11.45% for Zn-SL, and 9.77% for Zn-NR. Interestingly, the decrease was steeper in the first three cycles, followed by a flattening over the subsequent cycles (Fig. 12b), which is also observed in several other studies.^47,57,58^ This decrease is attributed to the change in the surface area of the catalyst, filling of its pores, and blockage of its active sites, as indicated by the N_2_ adsorption in the BET isotherm (Fig. 16a and b). However, the decrease is also attributed to the losses of the catalyst during its recovery after each cycle. Also, the crystalline structure of the catalysts remained intact over the multiple cycles, as confirmed by the pre- and post-use X-ray diffractograms of the three catalysts (ESI Fig. S4a and b,† respectively). This indicates the robustness of the catalyst and prepares the way for its commercial application.

**Fig. 17:**
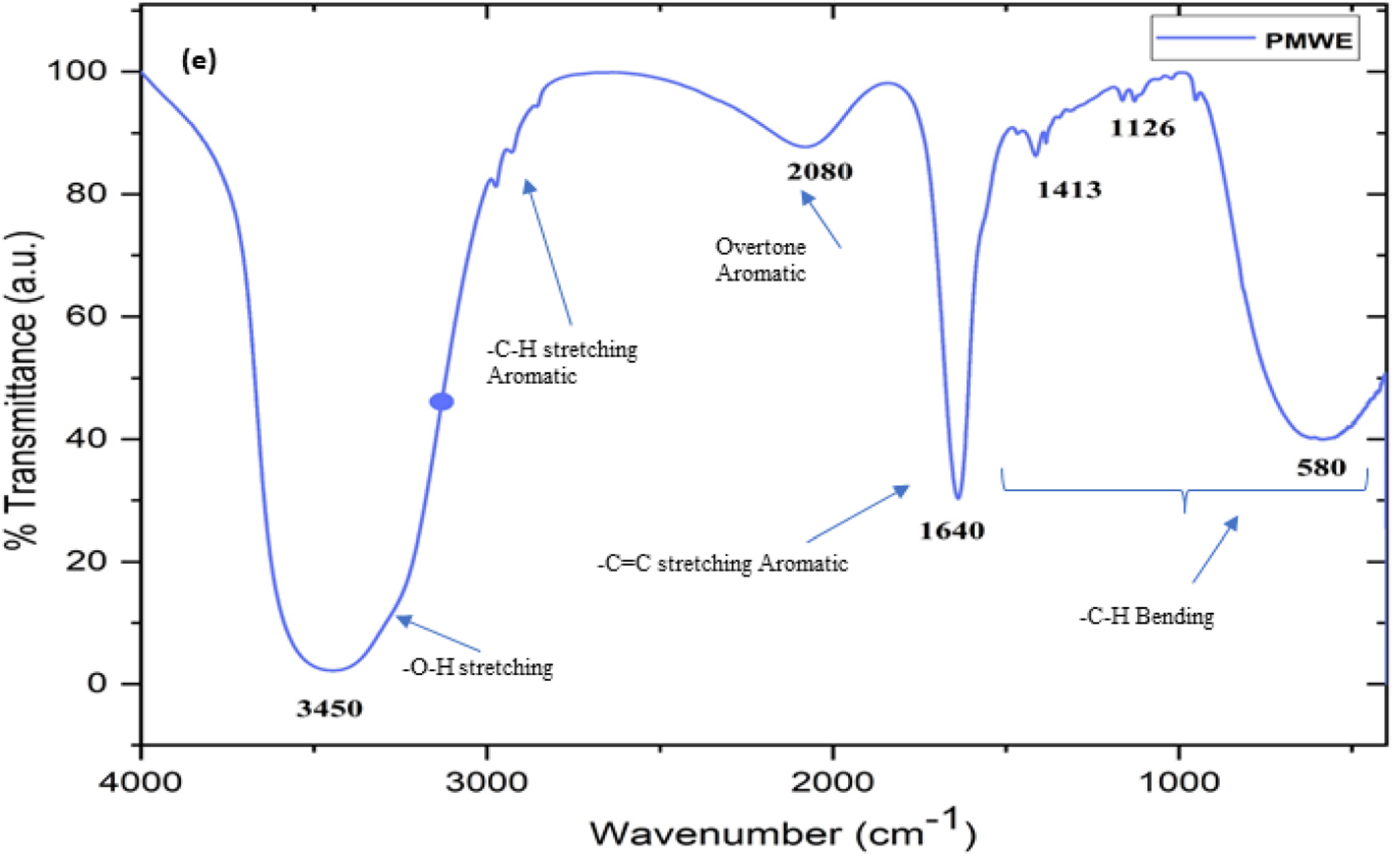
The Fourier-transform infrared spectrum (FTIR) of sugarcane press mud water extract (PMWE) with characteristic stretching peaks corresponding to the functional groups of the analyte present in the sample.

## 4 Conclusions

In summary, we synthesized the same three-phase (hexagonal, wurtzite *P*63*mc*) but morphologically different ZnO (ZN-AC, Zn-SL, and Zn-NR) NPs using an agro-waste (sugarcane press mud, PM)-based green sol–gel process. The interplay between the polyphenolic ligands present in the water extract of the agro-waste (PM) with the counter anion of the precursors seems to have controlled the size, morphology, and phase of the ZnO NPs by masking the screening effect posed by the counterions of the precursor salt. The application of these three catalysts at a low catalyst loading, *i.e.*, 0.1 g L^−1^, for the degradation of an organic pollutant (RhB dye) showed different rate constants (*k*), which is primarily due to the difference in their electrochemically active surface area and charge transfer barrier across catalyst–dye interface. However, this provides an avenue for the precursor neutral, phase-controlled, greener synthesis of ZnO and highlights the role of the catalyst–dye interface in photocatalyst design. Thus, this requires deeper and broader inquiries into green and controlled synthetic approaches considering the catalyst–dye interface to design broad-range photocatalysts delivering sustainable environmental solutions.

## Conflicts of interest

There are no conflicts to declare.

## Supplementary Material

NA-006-D3NA00596H-s001
