# Supplementary material for: Phase controlled green synthesis of wurtzite (P63mc) ZnO nanoparticles: interplay of green ligands with precursor anions, anisotropy and photocatalysis
Source: Nanoscale Adv. 2023 Nov 17;6(1):155–69. doi: 10.1039/d3na00596h (PMC10729870; doi:10.1039/d3na00596h)
Supplement: NA-006-D3NA00596H-s001 [file NA-006-D3NA00596H-s001.pdf]

Electronic Supplementary Material (ESI) for Nanoscale Advances.
This journal is © The Royal Society of Chemistry 2023

Supplementary information

For

Phase-controlled green synthesis of wurtzite ($P63_{mc}$) ZnO nanoparticles: Interplay of green ligands with precursor anions, anisotropy, and Photocatalysis

Lahur Mani Verma[1,2], Ajay Kumar[1], Aejaz Ul Bashir[2], Upanshu Gangwar Pravin P. Ingole[2], Satyawati Sharma[1*]

[1]CRDT, Indian Institute of Technology Delhi, New Delhi, India

[2]Department of Chemistry, Indian Institute of Technology Delhi, New Delhi, India

Corresponding author:

Dr. Satyawati Sharma

Email id: satyawatis@hotmail.com

List of contents: supplementary information file

Total number of pages (including cover): 11

Total number of figures: 5 *(the contributing figures labelled a, b, c, and d spread over different pages are part of respective figures labelled as 1,2,3 and 4)*

Total number of tables: 1

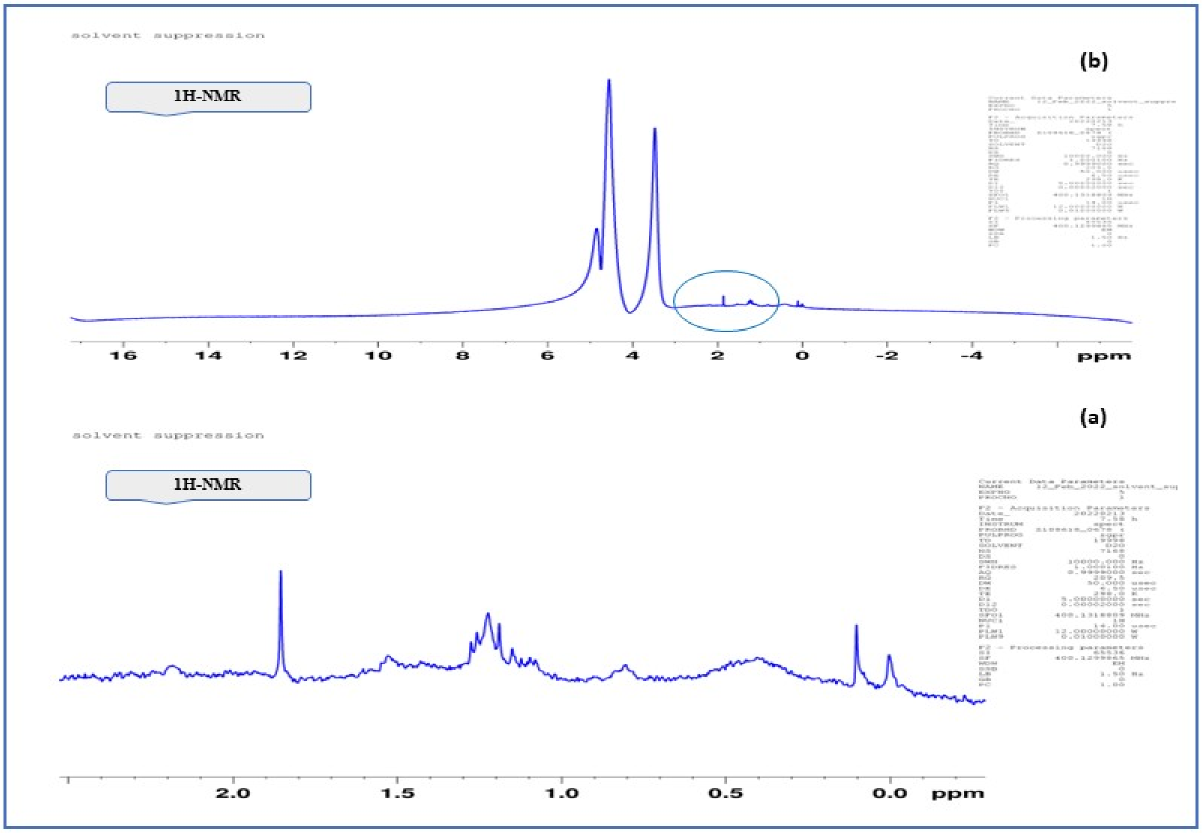
solvent suppression
(b)
1H-NMR
16 14 12 10 8 6 4 2 0 -2 -4 ppm
(a)
solvent suppression
1H-NMR
2.0 1.5 1.0 0.5 0.0 ppm

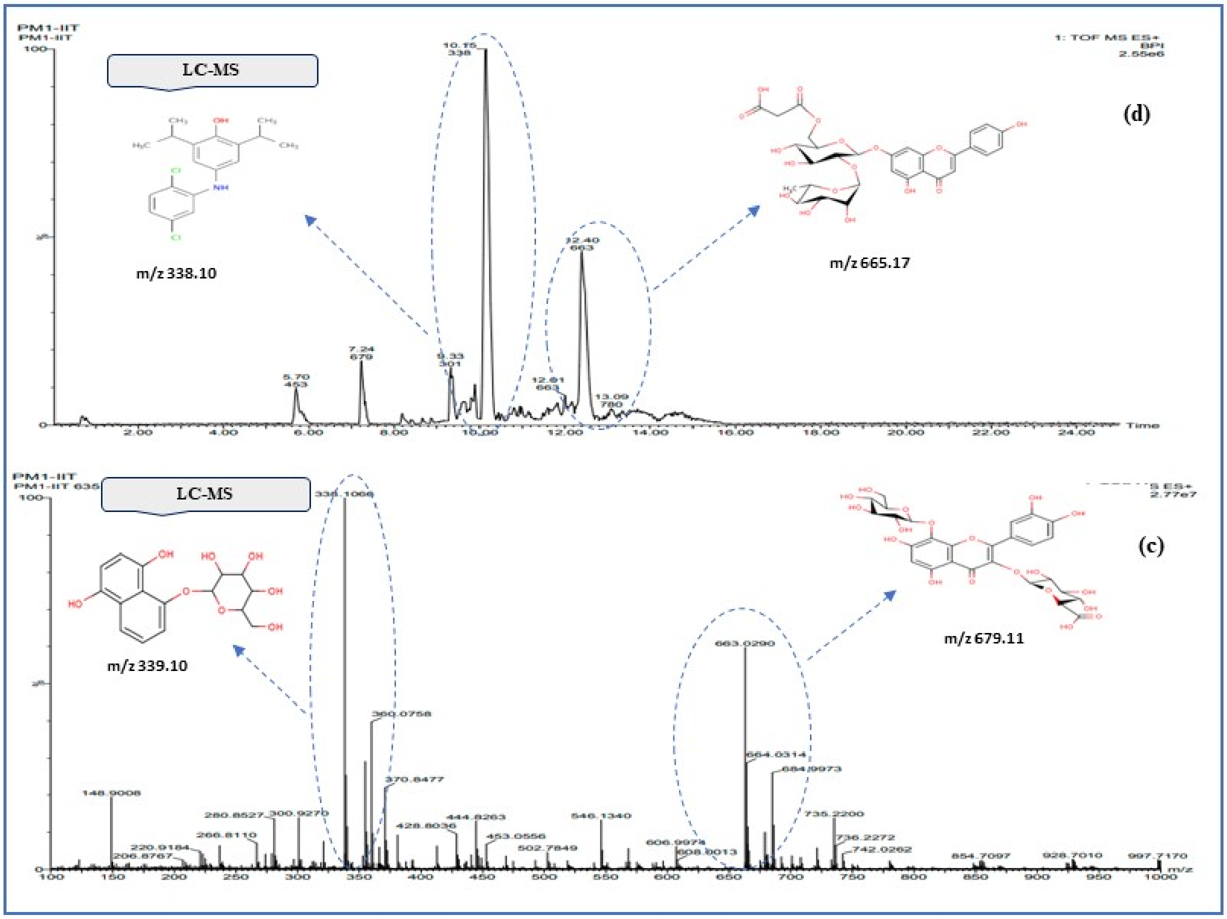

Fig. S1 (a-d) LC-MS Chromatogram and mass spectrum of sugar press mud water extract (c &d) the significant peaks at (retention time 10.15 and 12.40 min in chromatogram (c) corresponds the most probable compound (polyphenolic) in SPM water extract with $[M+H]^+$ peak having m/z 338.10/ 339.10 and m/z 679.11/665.17 respectively. H1NMR spectrum (a-b) with proton signal with chemical shifts in the range of (δ 0.0 -6.0 ppm) indicate Sp3 (C-H) and Sp2 (C-H), which support the m/z peaks of LC-MS for the probable compounds in the inset. The compound in the inset figure has been screened out based on m/z values from the mass library (Metlin.)

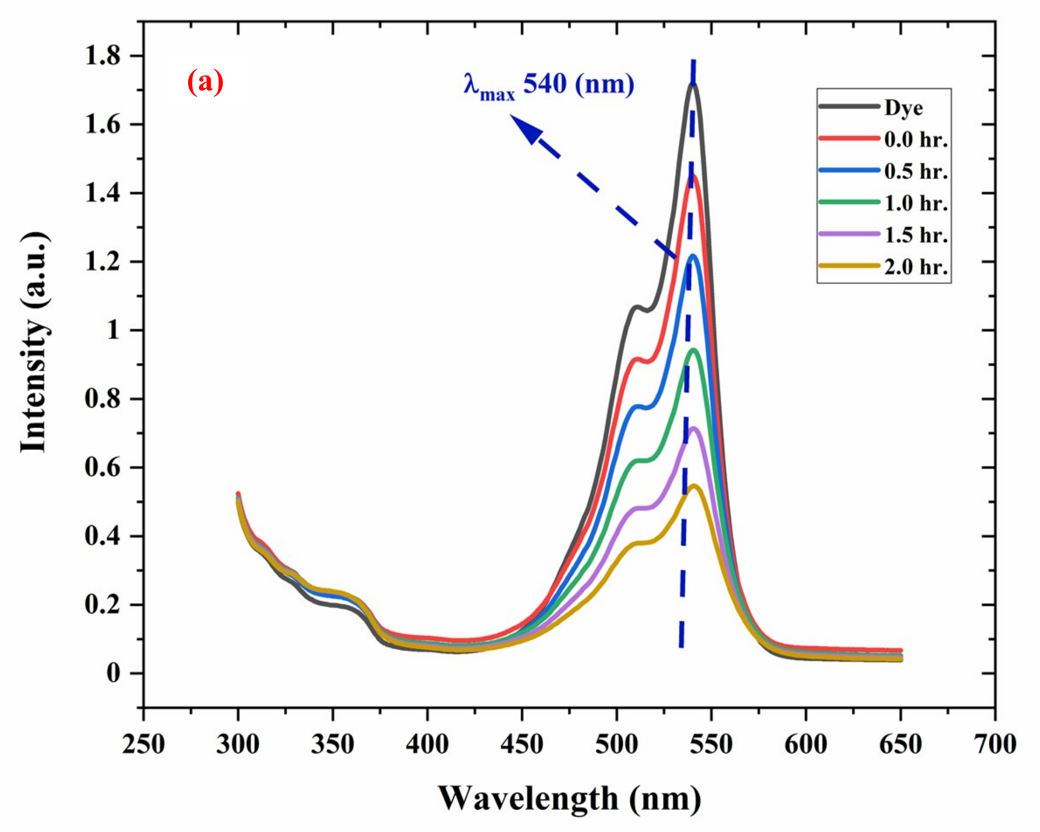

(a)
λmax 540 (nm)
Dye
0.0 hr.
0.5 hr.
1.0 hr.
1.5 hr.
2.0 hr.
Intensity (a.u.)
Wavelength (nm)
0
0.2
0.4
0.6
0.8
1
1.2
1.4
1.6
1.8
250
300
350
400
450
500
550
600
650
700

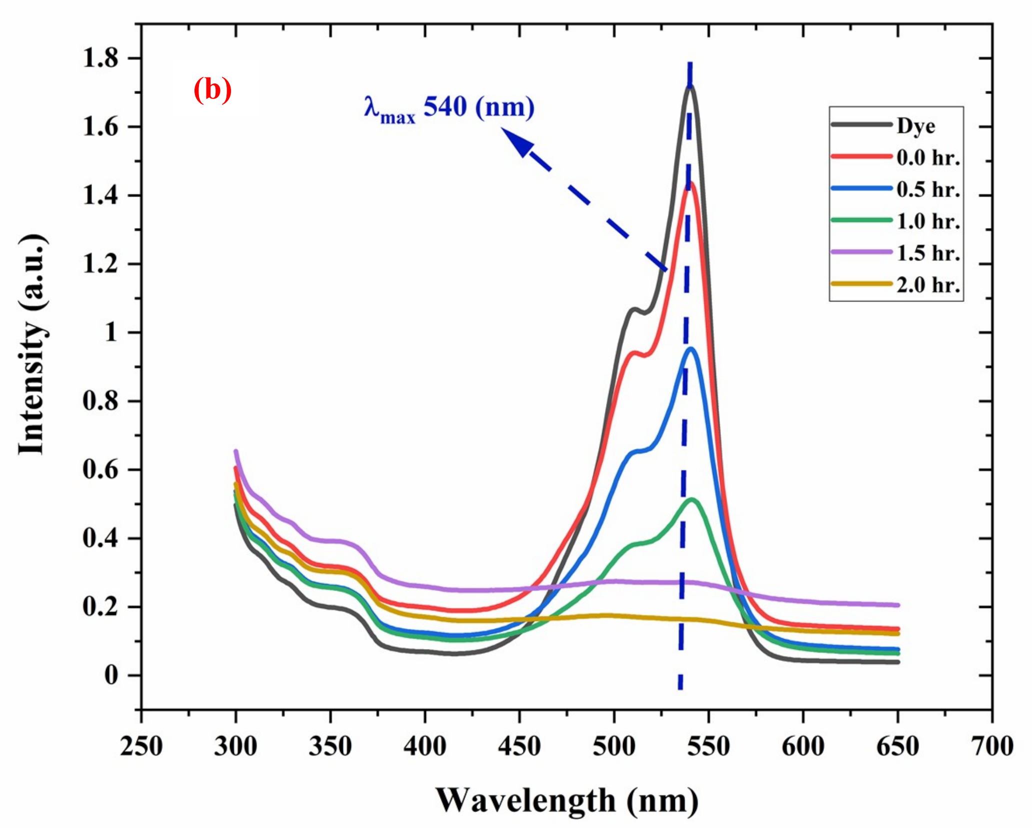

(b)
λmax 540 (nm)
Dye
0.0 hr.
0.5 hr.
1.0 hr.
1.5 hr.
2.0 hr.
Intensity (a.u.)
Wavelength (nm)

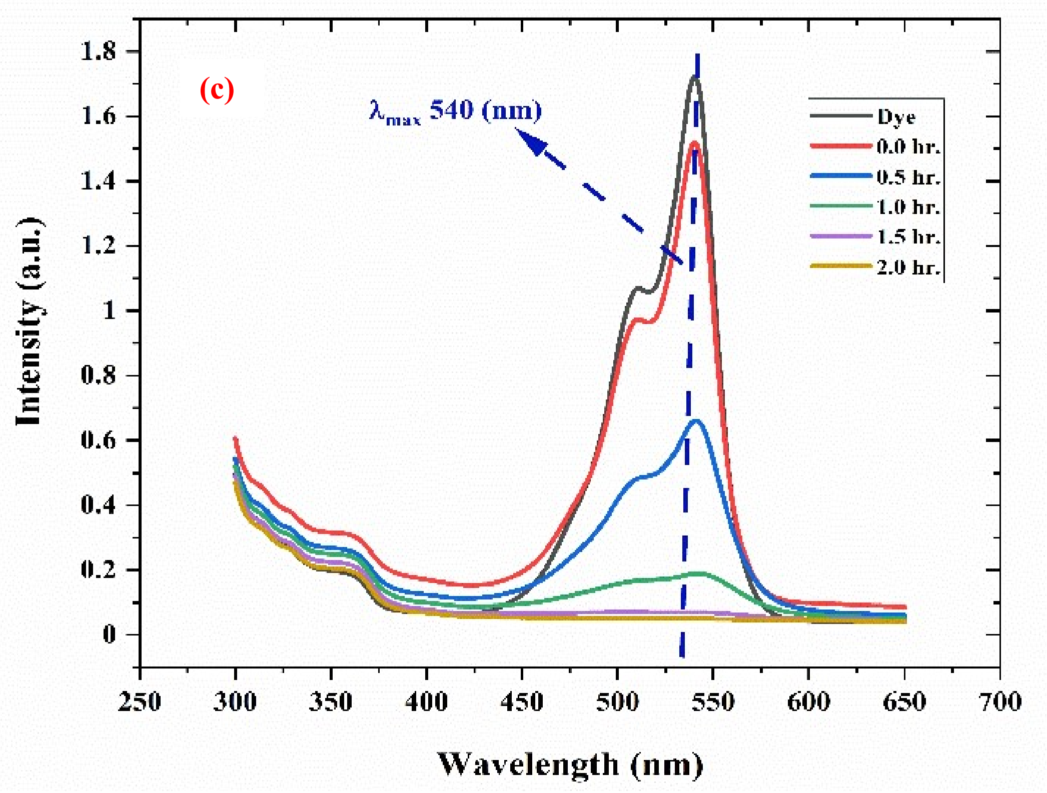

(c)
λmax 540 (nm)
Dye
0.0 hr.
0.5 hr.
1.0 hr.
1.5 hr.
2.0 hr.
Intensity (a.u.)
Wavelength (nm)

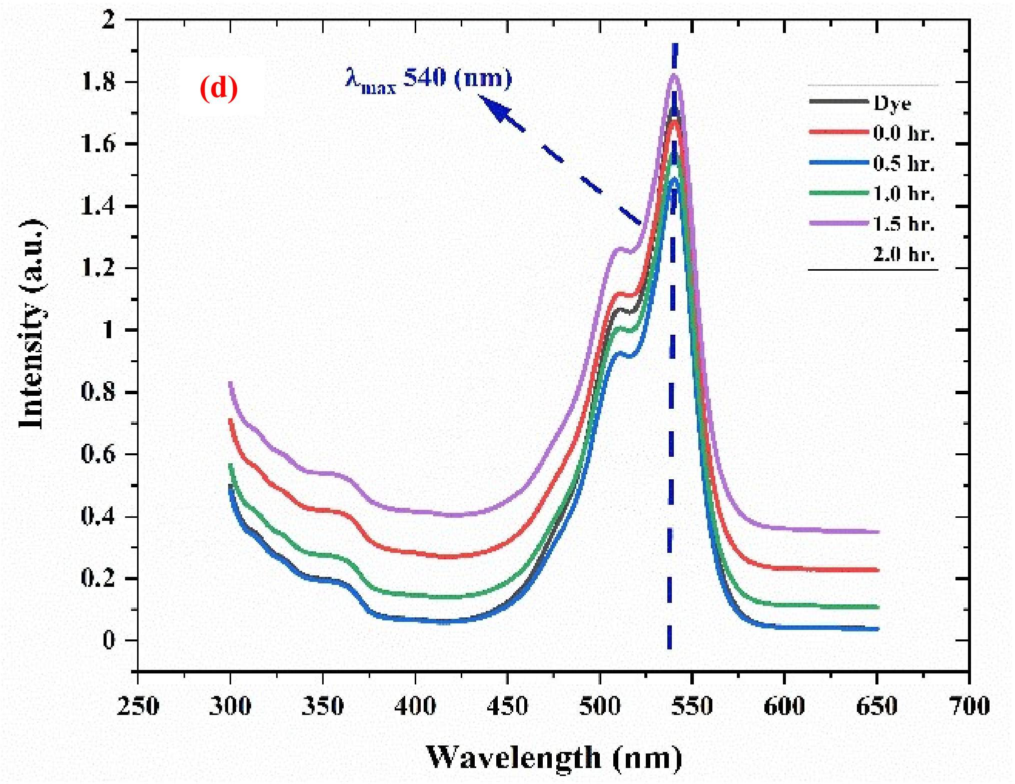

Fig. S2 (a-d) Uv- visible absorption spectrum of rhodamine dye (conc.0.596x 10^{-3}) degradation while testing the cyclic reusability of the catalyst of the different photocatalysts (conc. 0.9g/ L) suspension system under direct sunlight exposure recorded at the time interval of 0.5 hr. fig. Fig.11 (a) corresponds to Zn-NR catalyst system fig.11 (b) corresponds to Zn-SL catalyst system; like-wise 11(c) to Zn-AC; and fig.11 (d). control experiment (dye-catalyst system in the dark);

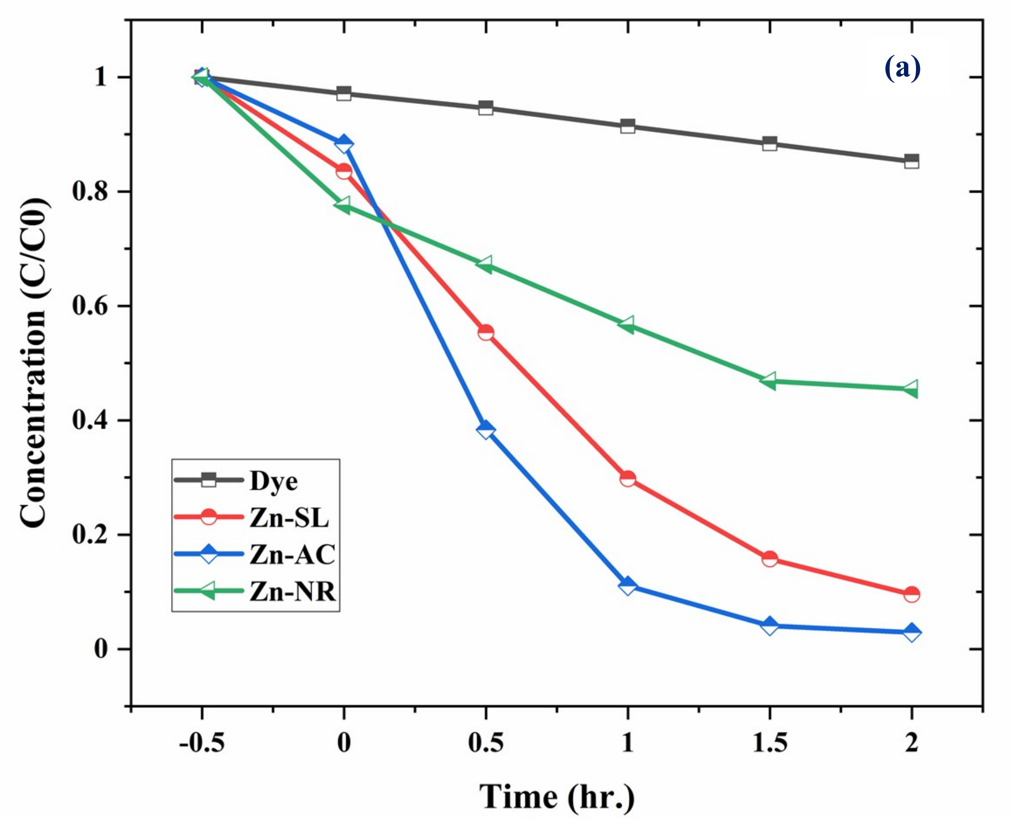
(a)
Concentration (C/C0)
Time (hr.)
Dye
Zn-SL
Zn-AC
Zn-NR
0
0.2
0.4
0.6
0.8
1
-0.5
0
0.5
1
1.5
2

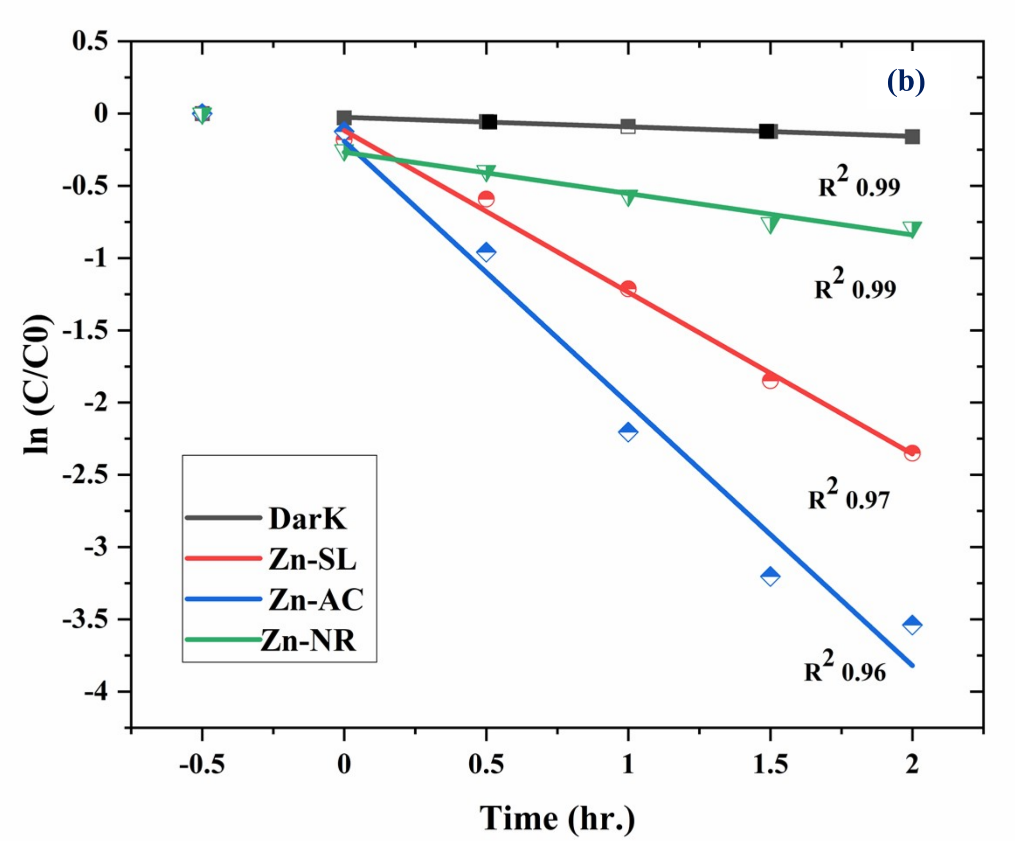

Fig. S3 (a-b) Concentration (C/C0) vs. time plot (fig.12 (a) showing a differential decrease in dye concentration with time for different catalyst systems. Fig.12 (b) plot (ln C/C0) vs time shows pseudo-first order kinetics with slope (rate constant 'k') in the range of (m= -0.06581 to -1.81453). with the R^2 values in the range of 0.95 to 0.99.

Table S1 The catalyst system and the corresponding rate constant for RhB dye degradation reaction.

Catalyst system	Rate constant (min^{-1})	AverageTemperature range (˚C)	Initial conc.(C_0)
**Dark**	-0.06581	34—37	0.596x10^{-3}
**Zn-SL**	-1.11947	34—37	0.596x10^{-3}
**Zn-AC**	-1.81453	34—37	0.596x10^{-3}
**Zn-NR**	-0.28606	34—37	0.596x10^{-3}

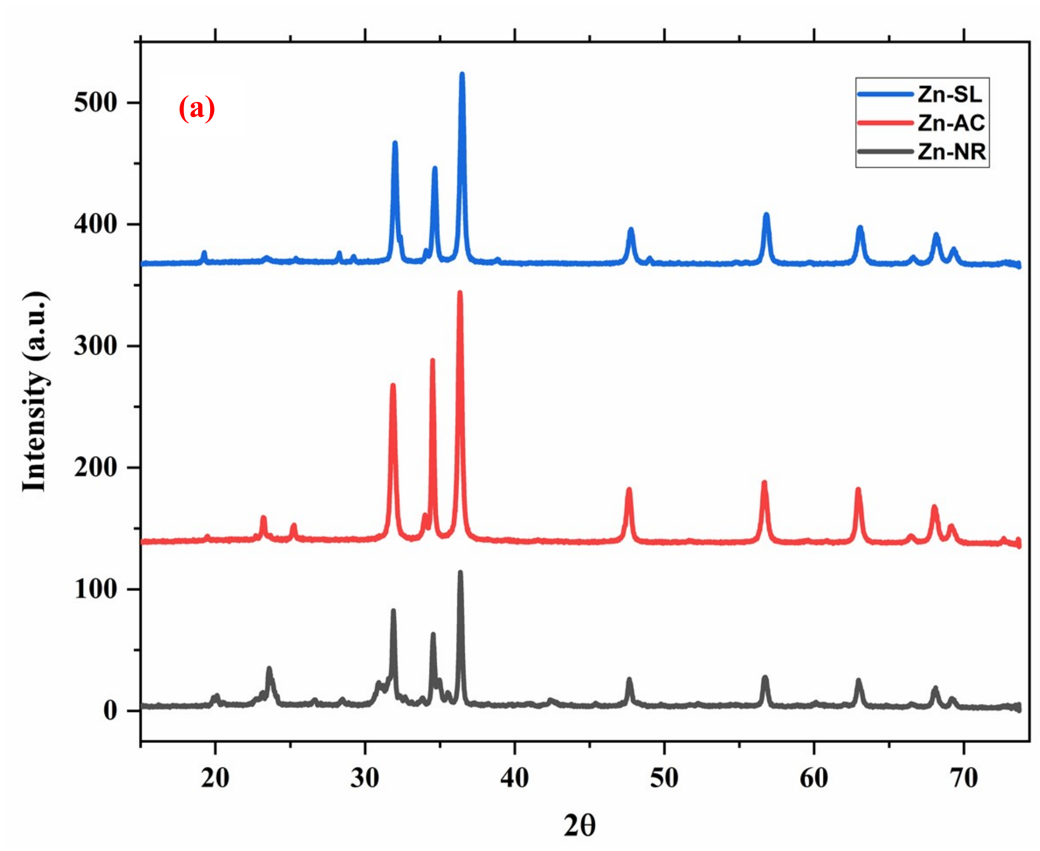
(a)
Zn-SL
Zn-AC
Zn-NR
Intensity (a.u.)
2θ
0
100
200
300
400
500
20
30
40
50
60
70

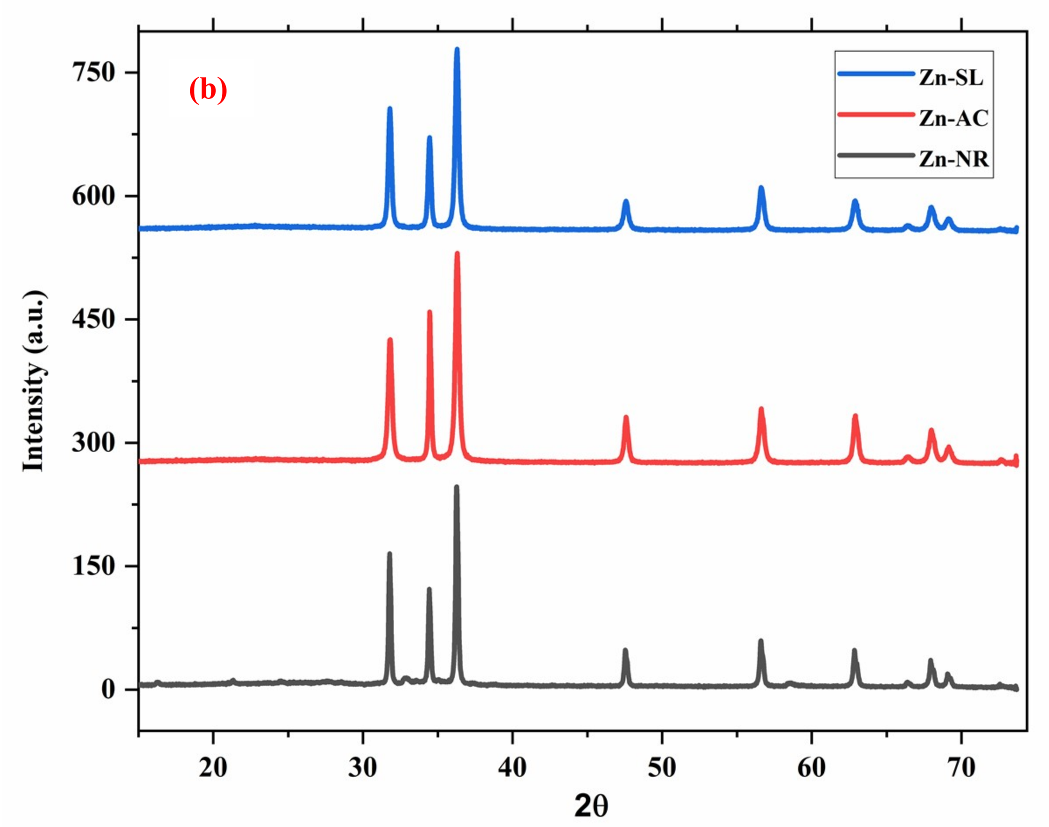

Fig. S4 (a-b) The x-ray diffractograms (a &b) of the three structurally different photocatalysts (NPs of ZnO); (a) diffractograms before the use of catalyst in photocatalysis; (b) After the use of the catalyst in photocatalysis; demonstrating structural integrity of the catalyst over repeated use.

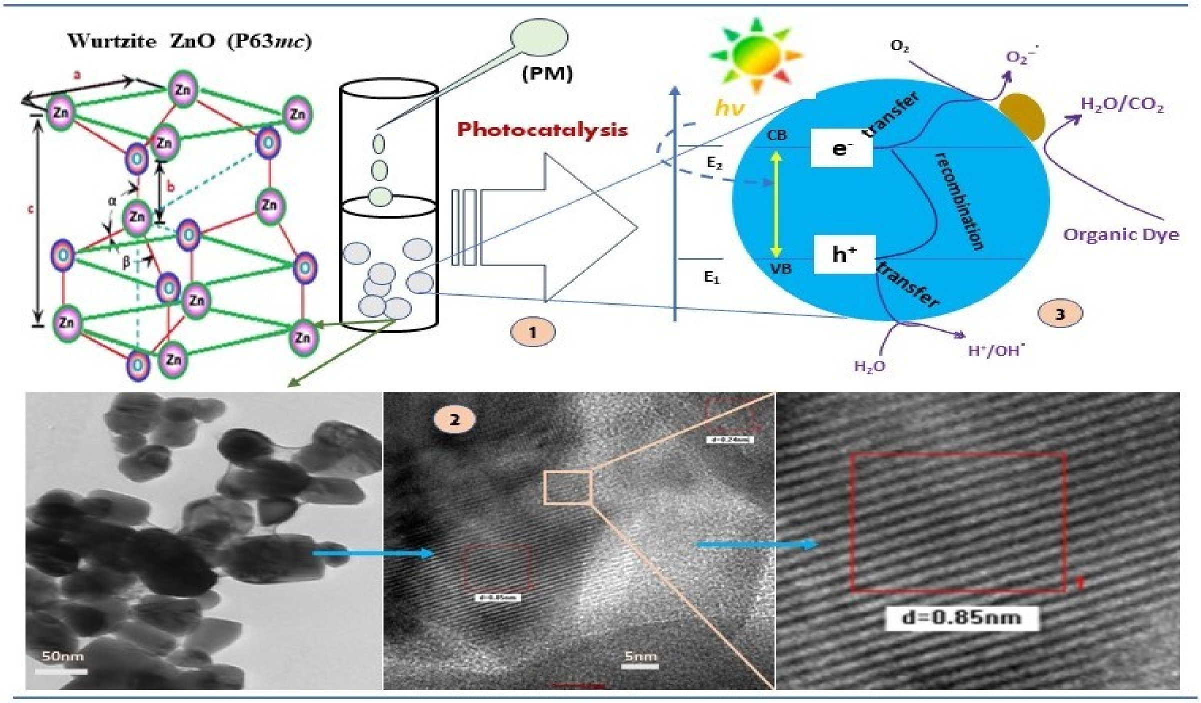

Fig. S5 schematic diagram describing synthesis, characterization and photocatalysis
